# Supplementary material for: Stress vulnerability promotes an alcohol‐prone phenotype in a preclinical model of sustained depression
Source: Addict Biol. 2018 Dec 18;25(1):e12701. doi: 10.1111/adb.12701 (PMC6916303; doi:10.1111/adb.12701)
Supplement: Supplementary file 1 — Figure S1. Development of affective and cognitive deficits in SDPS‐prone and SDPS‐resilient rats. Figure S2. Operant behavior during acquisition of alcohol self‐administration Figure S3. Active responding during re‐training in FR1 Figure S4. Extinction of operant alcohol self‐administration. Figure S5. Cumulative responding during operant alcohol self‐administration. Table S1. SDPS facilitates acquisition of operant alcohol self‐administration. Table S2. SDPS increases the number of rewards gained per training schedule. Table S3. SDPS increases operant alcohol intake. Table S4. SDPS does not affect general operant responding. Table S5. Alcohol intake at FRmax during PR sessions. Table S6. SDPS vulnerability increases non‐reinforced responding for alcohol. Table S7. SDPS vulnerability delays extinction learning. [file ADB-25-na-s001.docx]

Social defeat-induced persistent stress (SDPS)

Male Long-Evans rats, (Charles River, UK, weighing >500 g), paired-housed with age-matched tube-ligated females (Wistar, Harlan), were used for the resident-intruder protocol. Due to the large number of animals participating in the experiments, social defeat and subsequent behavioral assessment were performed in 3 consecutive weeks, as rats arrived in the vivarium in 3 independent batches, separated by 1 week each.

Assessment of the depressive-like state

Before participating in any behavioral measurement, all animals were transferred to the video-recording room and habituated to the test arena (plastic, opaque, 79 x 57 x 42 cm) for at least 10 minutes during 3 consecutive days. Animals were subjected to the Social Approach-Avoidance (SAA) and the Object Place Recognition (OPR) tasks at different time points, as indicated in Figure S1. All video recordings were analyzed with the Viewer^2^ software (BiObserve GmbH, Bonn, Germany).

*Social approach-avoidance test (SAA)* – Approach-avoidance behavior was estimated using an unfamiliar Long-Evans adult male rat (resident) as previously described^1, 2^. In order to examine the development and progression of social withdrawal the weeks after social defeat, all animals were exposed to 5 consecutive SAA tests: the week before social defeat (baseline -bl); following the defeat week (acute -w1); at week 5 (w5); at week 9 (w9) and at 6 months (6mth) following the last defeat exposure. In brief, rats were habituated and allowed to explore two empty target boxes (TBs, perforated, metal, 16 x 7 x 8 cm) located in the opposite sides of the testing arena (sample phase). Subsequently, an unfamiliar resident was introduced to one of the TBs and rats were allowed to explore and interact with the target, in absence of direct physical contact (test phase). Interaction index was calculated as time spent in active zone (resident zone)/ total exploration time (resident + neutral zone), during the first minute of a 5-minute test. Active and inactive zones were randomly assigned, in all tests provided and between groups, to avoid development of preference.

*Object place recognition (OPR)* – Hippocampal-dependent short-term memory was assessed by the object place recognition task using a 15-minute retention interval as previously described^1, 2^. In order to examine the development and progression of cognitive impairments after SDPS, all animals participated in three OPR tests, given the week before social defeat (baseline, bl), in week 5 (w5) and in week 9 (w9) following the last defeat exposure. In brief, following habituation, rats were allowed to explore two identical objects (cylinders or cubes, metal, 8 x 8 x 35 cm), located in two opposite corners of the arena (sample phase). After a 15-minute time interval, both objects were replaced with 2 identical ones, and one was displaced to a different position. Discrimination between the spatial locations of the two objects was used to assess spatial memory (exploration index = time spent in novel location / total exploration time (novel + familiar location)) during the first minute of a 3-minute test. The position of novel and familiar locations and the choice of object shapes were random in all tests provided and between groups, to avoid development of preference.

*Selection procedure* – For two-step cluster analysis the number of resulting clusters was determined automatically, to avoid a biased subject selection due to a fixed number of emerging subgroups. Both SAA and OPR were weighted equally for final group assignment, as the criterion for susceptibility or resilience required to include both affective and cognitive aspects of the depressive-like phenotype. First, we performed cluster analysis using the SAA data and SDPS animals were classified as prone or resilient based on their motivation to interact with the social target (affective deficits). Subsequently, OPR data were used for cluster analysis in order to identify prone *vs*. resilient individuals in respect to spatial memory retention (cognitive deficits). Animals that showed overlapping clustering in the two cluster analyses were finally identified as SDPS-prone (n = 15) and SDPS-resilient (n = 15). For further details, please see ^3^.

*Treatment administration* – We previously showed that pretreatment with guanfacine, an α_2_ adrenoreceptor agonist, ameliorates SDPS-induced excessive motivational drive, and normalizes reinstatement of alcohol-seeking in the SDPS-exposed general population^1^. To replicate these earlier findings, and verify its beneficial effects in the SDPS-prone animals, guanfacine-HCl (N-amidino-2-(2,6-dichlorophenyl) acetamide hydrochloride) was used on two separate cue-induced relapse tests. Saline (1 ml/kg) or guanfacine (0.5 mg/kg dissolved in saline) were systemically (i.p.) administered 1-h before each test in a cross-over design. A 72-h interval between the two tests was employed to ensure no carry-over effects of guanfacine.

Statistical analyses

All behavioral data collected from SAA, OPR tests were analyzed using repeated measures analysis of variance (ANOVA). When *P*-values reached level of significance (*P*<0.05), further analysis was performed using one-way ANOVA, paired or unpaired student’s t-test and post-hoc Tukey-HSD multiple comparisons. Homogeneity of variance was estimated and Hyunh-Feldt correction or non-parametric Kruskal-Wallis one-way ANOVA were implemented in case of assumption violation. Preference in interaction and exploration indexes (SAA, OPR) was estimated against a fictive group representing performance at chance levels, while retaining the same variation as the experimental groups^4^. All statistics were performed using IBM SPSS Statistics 24.

During assessment of the depressive-like state, the tracking software was erroneously terminated, thus datasets for the following tests were incomplete: ORR_bl_, n=2; OPR_w5_, n=3.

**Supplemental Figures**

**
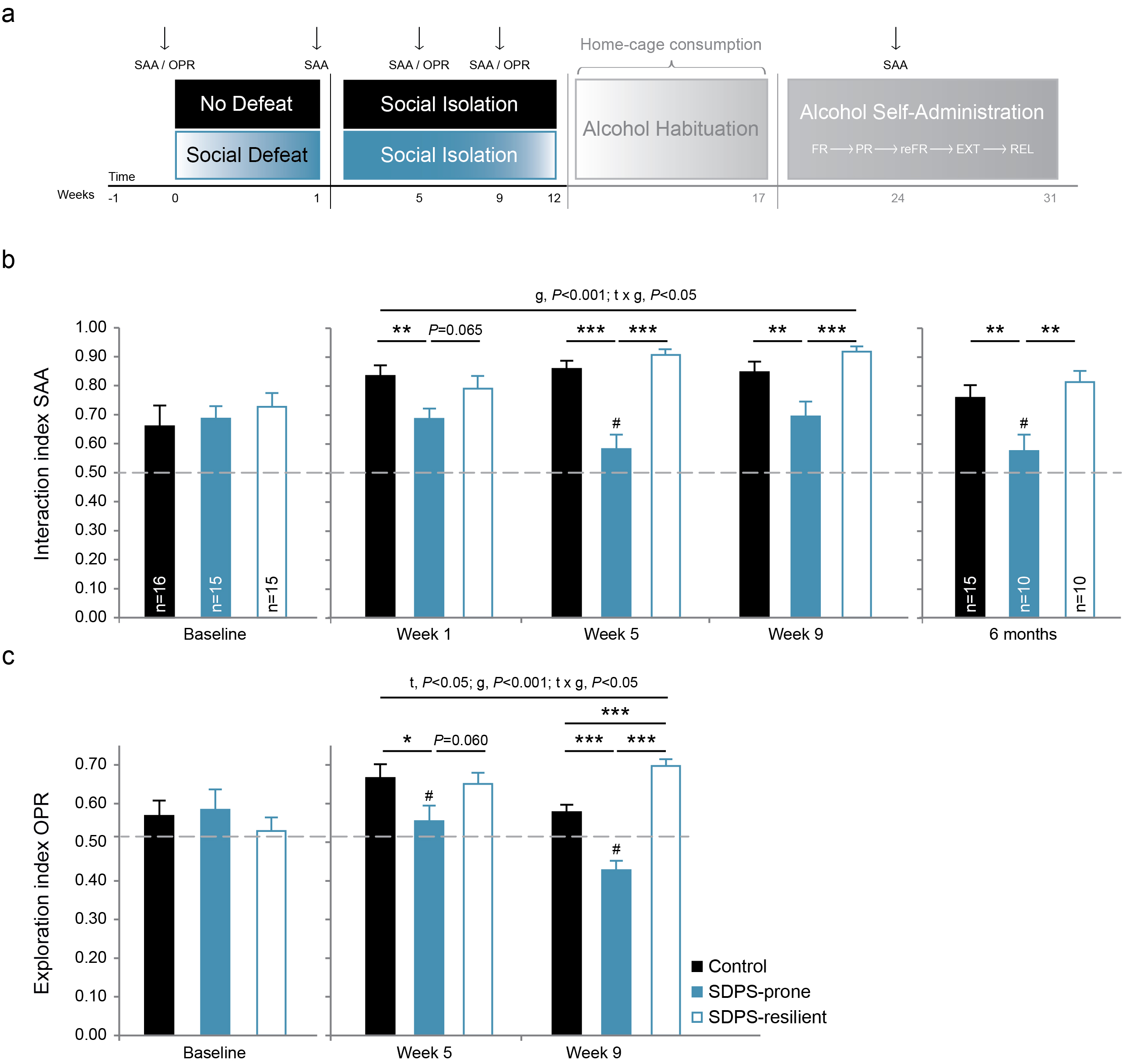
**

**Figure S1. Development of affective and cognitive deficits in SDPS-prone and SDPS-resilient rats.**

**a)** Experimental time-line. Wistar rats were exposed to 5 daily defeat sessions (week 0) and were subsequently single-housed for a period of ~6 months. The week before defeat (*w-1*), acutely after (*w1*), at 1 (*w5*), 2 (*w9*) and 6 months (*w24*) following defeat, SDPS effects in social approach-avoidance (SAA) and object place recognition (OPR) tasks were assessed. Based on SAA and OPR performance at weeks 5 and 9, SDPS rats were clustered into SDPS-prone or SDPS-resilient. **b)** Approach-avoidance behavior was examined in 5 SAA tests provided overtime. Before defeat, no preexisting differences were observed between the three groups (F(2,45)=0.36, *P*=0.700). In the months following defeat, significant group differences (F(2,43)=28.06, *P*<0.001) were observed, due to a decrease in interaction index in SDPS-prone rats (*P*<0.001 *vs.* both control and SDPS-resilient groups), indicative of the development of social withdrawal. No differences between control and SDPS-resilient groups were observed. In SDPS-prone rats, social avoidance persisted up to 6 months following the last defeat episode (F(2,34)=6.19, *P*=0.005; post-hoc: SDPS-prone *vs.* controls, *P*=0.008, *vs.* SDPS-resilient, *P*=0.002). SDPS-resilient animals showed normal social interaction (*P*=0.419 *vs.* controls). **c)** Short-term spatial memory was assessed in 3 OPR tests given overtime. Prior to defeat no between-group differences were observed F(2,43)=0.43, *P*=0.651. In the months following defeat, significant group differences (F(2,42)=17.73, *P*<0.001) were observed, due to a decrease in exploration index in SDPS-prone rats (*P*<0.001 *vs.* both control and SDPS-resilient groups), indicative of the development of cognitive deficits. Both control and SDPS-resilient groups displayed intact memory retention. Repeated measures ANOVA main time (t) and group (g) effect, time x group (t x g) interaction and pairwise comparisons are indicated. Dashed line represents chance levels (0.50) of interaction (SAA) or exploration (OPR). ^#^no preference for the social target (SAA) or the displaced object (OPR); **P*<0.05; ***P*<0.01; ****P*<0.001;

**
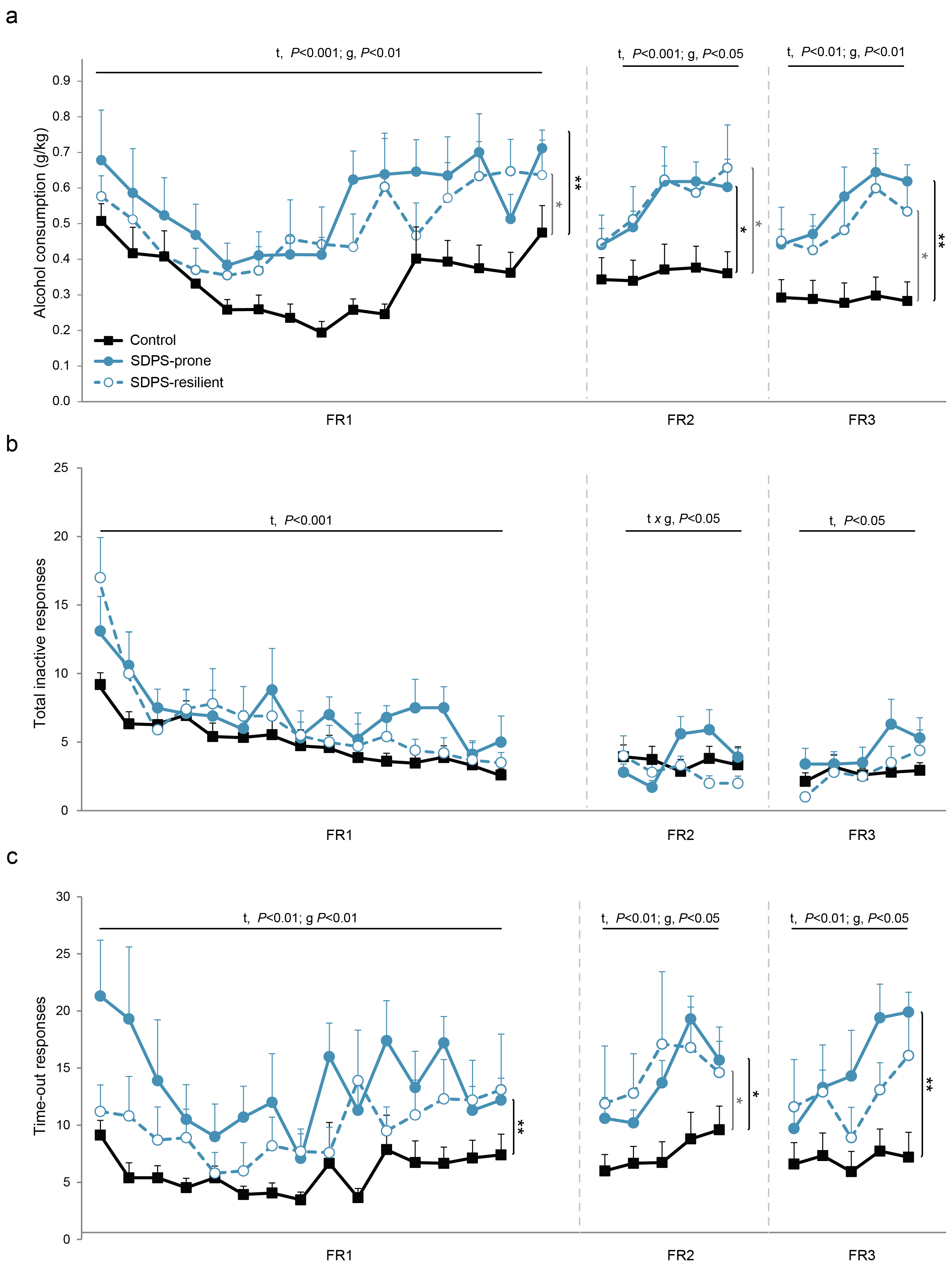
**

**Figure S2. Operant behavior during acquisition of alcohol self-administration**

After habituation in the home-cage, all animals were trained to respond for an alcohol reward under fixed reinforcement schedules (FR1-3). **a)** Significant effects of training were seen in all three FRs, as alcohol consumption was adjusted in response to changes in reinforcement schedules. Similar to the number of rewards per session, both SDPS groups consumed significantly more alcohol as compared with controls, in all three FR schedules provided, while no group differences were seen between the two SDPS groups (Table S3). **b)** A significant training effect was observed for inactive responses in FR1, as all animals reduced responding to the inactive hole, indicating consolidation of the task, i.e., preference for the active, alcohol-associated hole (Table S4). Next, in FR2, a training x group interaction effect was observed, representing opposite direction of inactive responding in the two SDPS groups. A training effect was seen for FR3, as the two SDPS groups slightly increased their responses, reflecting changes in the reinforcement schedule. No between-group differences were observed in any of the schedules provided, illustrating that SDPS had no gross effects in rats’ discriminative ability (active *vs.* inactive hole) or general motoric skills. **c)** Analysis of time-out (non-reinforced) responses during all three reinforcement schedules revealed significant time and group effects, with the latter mainly driven by increased responding in the SDPS-prone rats when compared to controls (Table S6). No difference between the two SDPS subpopulations was observed. Although not corrected for increased active responding after SDPS, time-out data pinpointed to an SDPS effect on alcohol-seeking during unavailability periods, as function of the magnitude of the depressive-like state. Repeated measures ANOVA main time (t) and group (g) effects and pairwise comparisons (vertical lines, black, SDPS-prone *vs.* controls; grey, SDPS-resilient *vs.* controls) are indicated; **P*<0.05; ***P*<0.01.

**
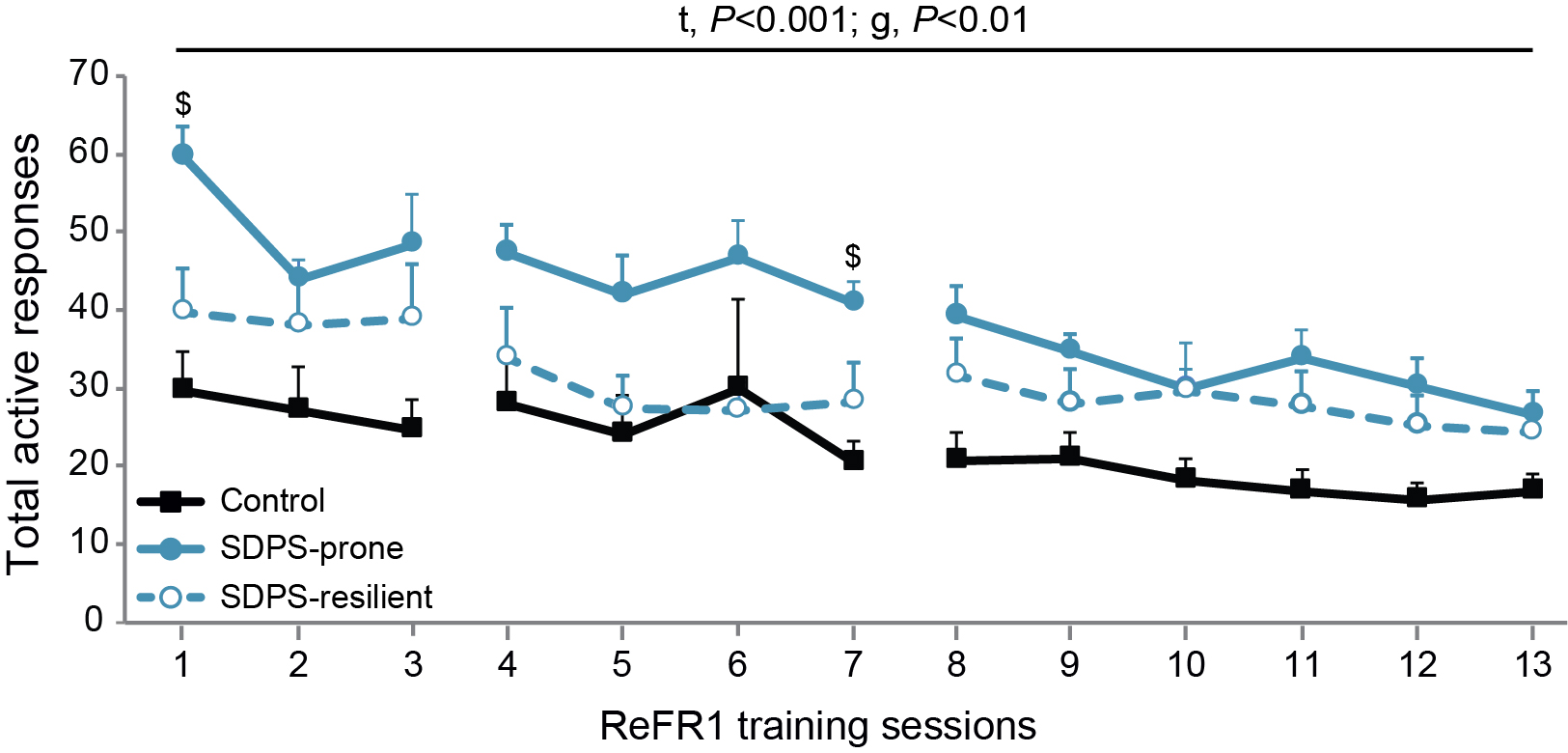
**

**Figure S3. Active responding during re-training in FR1**

Following PR schedules, and before extinction of the alcohol-associated context, all animals were subjected to 13 reFR1 training sessions. All rats reduced active responding, as the less demanding training schedule was introduced. Similar to initial acquisition in FR1, a significant group effect was observed, as SDPS-prone rats reached higher number of responses when compared with controls (*P*=0.002). The 4 training sessions (4-7) with increased time-out interval are depicted separately. Repeated measures ANOVA main time (t), group (g) and time x group (t x g) effects are indicated. ^$^significant difference between SDPS-prone *vs.* both SDPS-resilient and control groups.

**
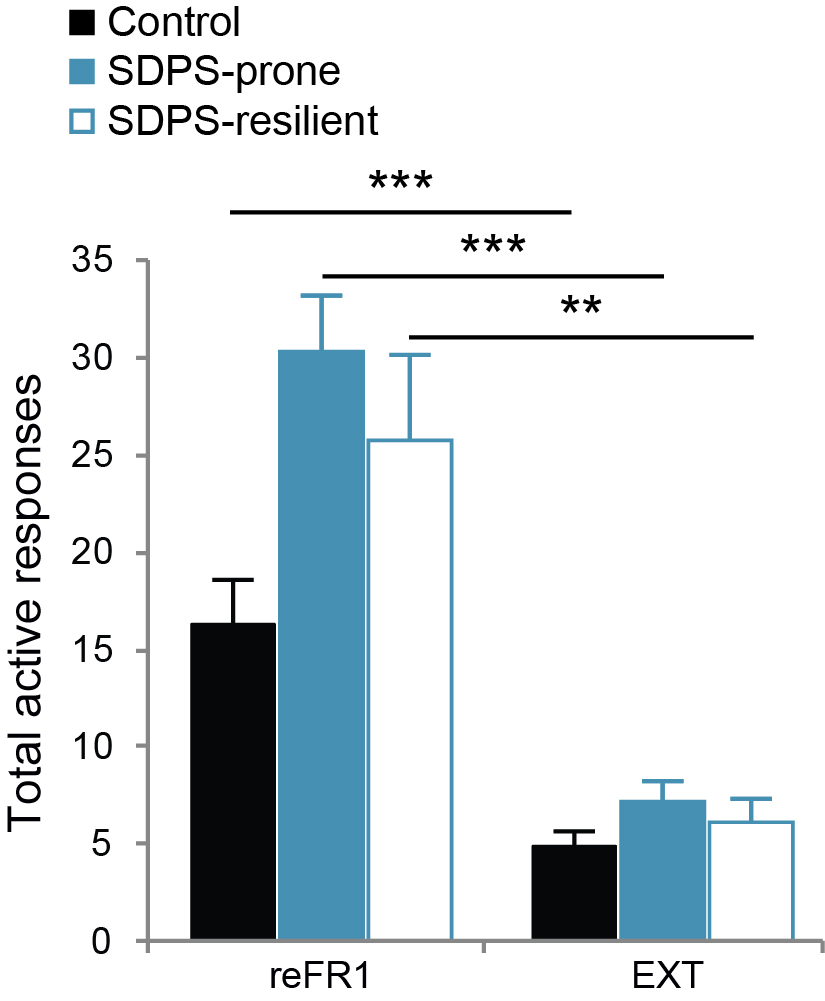
**

**Figure S4. Extinction of operant alcohol self-administration.**

Following re-training in FR1, all animals were subjected to 15 1-h extinction sessions, during which alcohol availability was omitted (*c.f.* Figure 5). Group performance at reFR1 and extinction training (average responses of last 3 sessions, respectively) is depicted here. All groups showed significant reduction in responding for an alcohol reward: Controls, t(14)=4.74, *P*<0.001; SDPS-prone, t(9)=6.71, *P*<0.001; and SDPS-resilient, t(9)=4.23, *P*=0.002. By the end of the extinction period, all groups were successfully extinguished responding to the alcohol-associated hole, as illustrated by them reaching less than 1/3 of their original responses during reFR1 (control, 4.9±0.7; SDPS-prone, 7.2±1.0; and SDPS-resilient, 6.1±1.2). Paired t-tests are indicated; **P*<0.05; ***P*<0.01.

**
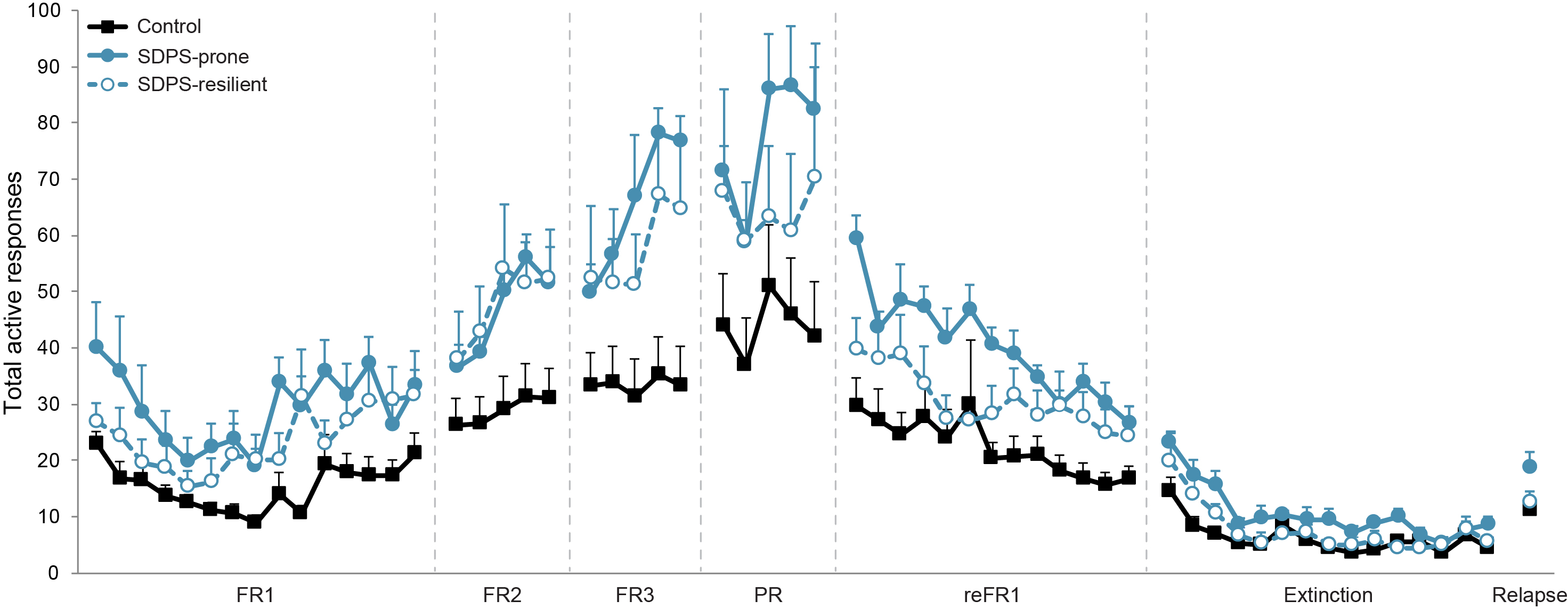
**

**Figure S5. Cumulative responding during operant alcohol self-administration.**

The total number of active responses per group is depicted during the whole period of alcohol self-administration in the operant chambers. Rats adapted their responding for an alcohol reward based on the training schedule of the different phases of SA. A general effect of SDPS is apparent, with both SDPS groups increasing responding versus controls during initial acquisition of alcohol SA (FR1-3). From PR training onwards, SDPS-prone animals acquired elevated number of responses versus their resilient counterparts, an effect that was maintained during extinction and reinstatement of alcohol SA.

**Supplemental Tables**

|  | *FR1* | *FR2* | *FR3* |
| --- | --- | --- | --- |
| **Training** | F(6.84,219.03)=5.05  ***P*<0.001** | F(2.64,84.61)=8.47  ***P*<0.001** | F(3.43,109.72)=5.02  ***P*=0.002** |
| **Group** | F(2,32)=7.32  ***P*=0.002** | F(2,32)=3.95  ***P*=0.029** | F(2,32)=7.11  ***P*=0.003** |
| **Training x Group** | F(13.69,219.03)=0.91  *P*=0.547 | F(5.29,84.61)=1.14  *P*=0.348 | F(6.86,109.72)=1.62  *P*=0.138 |
| **Post-Hoc** |  | | |
| Con vs. SDPS-prone | ***P*=0.001** | ***P*=0.029** | ***P*=0.001** |
| Con vs. SDPS-resilient | ***P*=0.032** | ***P*=0.021** | ***P*=0.013** |
| SDPS-prone vs. SDPS-resilient | *P*=0.183 | *P*=0.901 | *P*=0.413 |

**Table S1. SDPS facilitates acquisition of operant alcohol self-administration.**

Repeated measures ANOVA was employed to analyze alcohol acquisition during self-administration in the operant chambers. Fifteen FR1, five FR2 and five FR3 sessions were provided, and responding in the active hole was analyzed per group (SDPS-prone, n=10, SDPS-resilient, n=10; controls, n=15). Statistics for main training and group effects, as well as training x group interaction for each reinforcement schedule are summarized. Pairwise comparisons among the three groups and correspondent *P*-values are reported.

|  | *FR1* | *FR2* | *FR3* |
| --- | --- | --- | --- |
| **Training** | F(7.62,243.94)=7.26  ***P*<0.001** | F(3.00,96.02)=7.62  ***P*<0.001** | F(3.47,111.05)=4.56  ***P*=0.003** |
| **Group** | F(2,32)=6.16  ***P*=0.005** | F(2,32)=3.74  ***P*=0.035** | F(2,32)=7.06  ***P*=0.003** |
| **Training x Group** | F(15.25,243.94)=1.18  *P*=0.283 | F(6.00,96.02)=1.29  *P*=0.270 | F(6.94,111.5)=1.38  *P*=0.219 |
| **Post-Hoc** |  | | |
| Con vs. SDPS-prone | ***P*=0.002** | ***P*=0.031** | ***P*=0.001** |
| Con vs. SDPS-resilient | ***P*=0.025** | ***P*=0.027** | ***P*=0.011** |
| SDPS-prone vs. SDPS-resilient | *P*=0.375 | *P*=0.961 | *P*=0.474 |

**Table S2. SDPS increases the number of rewards gained per training schedule.**

Repeated measures ANOVA was employed to examine alcohol consumption in the operant chambers. Fifteen FR1, five FR2 and five FR3 sessions were provided, and the total number of rewards gained was analyzed per group (SDPS-prone, n=10, SDPS-resilient, n=10; controls, n=15). Statistics for main training and group effects, as well as training x group interaction for each reinforcement schedule are summarized. Pairwise comparisons among the three groups and correspondent *P*-values are reported.

|  | *FR1* | *FR2* | *FR3* |
| --- | --- | --- | --- |
| **Training** | F(7.75,247.89)=6.68  ***P*<0.001** | F(2.98,95.45)=7.42  ***P*<0.001** | F(3.49,111.65)=4.38  ***P*=0.004** |
| **Group** | F(2,32)=5.92  ***P*=0.006** | F(2,32)=3.78  ***P*=0.033** | F(2,32)=7.40  ***P*=0.002** |
| **Training x Group** | F(15.59,247.89)=1.14  *P*=0.321 | F(5.96,95.45)=1.42  *P*=0.216 | F(6.98,111.65)=1.40  *P*=0.214 |
| **Post-Hoc** |  | | |
| Con vs. SDPS-prone | ***P*=0.003** | ***P*=0.032** | ***P*=0.001** |
| Con vs. SDPS-resilient | ***P*=0.024** | ***P*=0.024** | ***P*=0.008** |
| SDPS-prone vs. SDPS-resilient | *P*=0.438 | *P*=0.911 | *P*=0.528 |

**Table S3. SDPS increases operant alcohol intake.**

Repeated measures ANOVA was employed to examine alcohol consumption in the operant chambers. Fifteen FR1, five FR2 and five FR3 sessions were provided, and the total ethanol intake, corrected for body weight, was analyzed per group (SDPS-prone, n=10, SDPS-resilient, n=10; controls, n=15). Statistics for main training and group effects, as well as training x group interaction for each reinforcement schedule are summarized. Pairwise comparisons among the three groups and correspondent *P*-values are reported.

|  | *FR1* | *FR2* | *FR3* |
| --- | --- | --- | --- |
| **Training** | F(9.27,296.71)=9.50  ***P*<0.001** | F(3.38,108.28)=1.25  *P*=0.294 | F(4,128)=3.23  ***P*=0.014** |
| **Group** | F(2,32)=2.51  *P*=0.097 | F(2,32)=0.59  *P*=0.561 | F(2,32)=1.85  *P*=0.174 |
| **Training x Group** | F(18.54,296.71)=0.89  *P*=0.588 | F(6.77, 108.28)=2.47  ***P*=0.023** | F(8,128)=0.88  *P*=0.532 |

**Table S4. SDPS does not affect general operant responding.**

Repeated measures ANOVA was employed to examine general psychomotor activity and task consolidation during alcohol self-administration in the operant chambers. Fifteen FR1, five FR2 and five FR3 sessions were provided, and the total number of inactive (with no apparent consequences) responses was analyzed per group (SDPS-prone, n=10, SDPS-resilient, n=10; controls, n=15). Statistics for main training and group effects, as well as training x group interaction for each reinforcement schedule are summarized, and correspondent *P*-values are reported.

|  | **PR1** | **PR2** | **PR3** | **PR4** | **PR5** |
| --- | --- | --- | --- | --- | --- |
| Control | 0.18±0.02 | 0.17±0.02 | 0.19±0.02 | 0.17±0.02 | 0.16±0.02 |
| SDPS-prone | 0.24±0.01* | 0.22±0.01* | 0.25±0.02* | 0.26±0.01* | 0.24±0.01* |
| SDPS-resilient | 0.21±0.03 | 0.21±0.02 | 0.21±0.03 | 0.20±0.02 | 0.21±0.03 |

**Table S5. Alcohol intake at FRmax during PR sessions.**

Total alcohol intake corrected for body weight, recorded over the 5 PR sessions. SDPS-prone animals consumed significantly more alcohol when compared with controls in all 5 sessions provided. Group mean ± SEM in g/kg is indicated. * *P*<0.05 *vs.* controls.

|  | *FR1* | *FR2* | *FR3* |
| --- | --- | --- | --- |
| **Training** | F(7.14,228.46)=2.89,  ***P*=0.006** | F(2.82,77.99)=4.54,  ***P*=0.006** | F(3.51,112.23)=4.31,  ***P*=0.004** |
| **Group** | F(2,32)=6.80,  ***P*=0.003** | F(2,32)=3.38  ***P*=0.046** | F(2,32)=4.81  ***P*=0.015** |
| **Training x Group** | F(14.28,228.46)=0.96,  *P*=0.496 | F(5.64,77.99)=0.85,  *P*=0.527 | F(7.01,112.23)=1.54, *P*=0.162 |
| **Post-Hoc** |  | | |
| Con vs. SDPS-prone | ***P*=0.001** | ***P*=0.049** | ***P*=0.005** |
| Con vs. SDPS-resilient | *P*=0.068 | ***P*=0.029** | *P*=0.056 |
| SDPS-prone vs. SDPS-resilient | *P*=0.116 | *P*=0.829 | *P*=0.369 |

**Table S6. SDPS vulnerability increases non-reinforced responding for alcohol.**

Repeated measures ANOVA was employed to examine alcohol-seeking during time-out periods. Fifteen FR1, five FR2 and five FR3 sessions were provided, and the total number of non-reinforced responses to the active hole was analyzed per group (SDPS-prone, n=10, SDPS-resilient, n=10; controls, n=15). Statistics for main training and group effects, as well as training x group interaction for each reinforcement schedule are summarized. Pairwise comparisons among the three groups and correspondent *P*-values are reported.

|  | *EXT1-5* | *EXT6-10* | *EXT11-15* |
| --- | --- | --- | --- |
| **Time** | F(3.23,103.41)=23.58  ***P*<0.001** | F(3.62,115.84)=4.87  ***P*=0.002** | F(3.69,117.98)=4.14  ***P*=0.005** |
| **Group** | F(2,32)=6.65  ***P*=0.004** | F(2,32)=4.61  ***P*=0.017** | F(2,32)=2.35  *P*=0.112 |
| **Time x Group** | F(6.46,103.41)=0.72  *P*=0.647 | F(7.24,115.84)= 0.74  *P*=0.646 | F(7.37,117.98)=1.64  *P*=0.127 |
| **Post-Hoc** |  | | |
| Con vs. SDPS-prone | ***P*=0.001** | ***P*=0.006** | n/a |
| Con vs. SDPS-resilient | *P*=0.097 | *P*=0.573 | n/a |
| SDPS-prone vs. SDPS-resilient | *P*=0.089 | ***P*=0.036** | n/a |

**Table S7. SDPS vulnerability delays extinction learning.**

Repeated measures ANOVAs were employed to analyze extinction of alcohol-seeking. Given an overall time effect (*cf.* Fig. 5b) in active responding, extinction performance was analyzed in 3 bins of 5 extinction sessions, representing each week of training in-between no-training weekend days. Statistics for main time and group effects, as well as time x group interaction for each training bin are summarized. Pairwise comparisons for the main group effect and correspondent *P*-values are reported.

**References**

1. Riga D, Schmitz LJ, van der Harst JE, van Mourik Y, Hoogendijk WJ, Smit AB*, et al*. A sustained depressive state promotes a guanfacine reversible susceptibility to alcohol seeking in rats. *Neuropsychopharmacology : official publication of the American College of Neuropsychopharmacology* 2014; **39**(5)**:** 1115-1124.

2. Riga D, Theijs JT, De Vries TJ, Smit AB, Spijker S. Social defeat-induced anhedonia: effects on operant sucrose-seeking behavior. *Front Behav Neurosci* 2015; **9:** 195.

3. Riga D, Schmitz LJM, Hoogendijk WJG, Smit AB, Spijker S. Temporal profiling of depression vulnerability in a preclinical model of sustained depression. *Sci Rep* 2017; **7**(1)**:** 8570.

4. Akkerman S, Prickaerts J, Steinbusch HW, Blokland A. Object recognition testing: statistical considerations. *Behavioural brain research* 2012; **232**(2)**:** 317-322.
